# Expression and water calcium dependence of calcium transporter isoforms in zebrafish gill mitochondrion-rich cells

**DOI:** 10.1186/1471-2164-8-354

**Published:** 2007-10-04

**Authors:** Bo-Kai Liao, Ang-Ni Deng, Shyh-Chi Chen, Ming-Yi Chou, Pung-Pung Hwang

**Affiliations:** 1 Institute of Cellular and Organismic Biology, Academia Sinica, Taipei, Taiwan, ROC; 2 Institute of Fisheries Science, National Taiwan University, Taipei, Taiwan, ROC

## Abstract

**Background:**

Freshwater fish absorb Ca^2+ ^predominantly from ambient water, and more than 97% of Ca^2+ ^uptake is achieved by active transport through gill mitochondrion-rich (MR) cells. In the current model for Ca^2+ ^uptake in gill MR cells, Ca^2+ ^passively enters the cytosol via the epithelium Ca^2+ ^channel (ECaC), and then is extruded into the plasma through the basolateral Na^+^/Ca^2+ ^exchanger (NCX) and plasma membrane Ca^2+^-ATPase (PMCA). However, no convincing molecular or cellular evidence has been available to support the role of specific PMCA and/or NCX isoforms in this model. Zebrafish (*Danio rerio*) is a good model for analyzing isoforms of a gene because of the plentiful genomic databases and expression sequence tag (EST) data.

**Results:**

Using a strategy of BLAST from the zebrafish genome database (Sanger Institute), 6 isoforms of PMCAs (PMCA1a, PMCA1b, PMCA2, PMCA3a, PMCA3b, and PMCA4) and 7 isoforms of NCXs (NCX1a, NCX1b, NCX2a, NCX2b, NCX3, NCX4a, and NCX4b) were identified. In the reverse-transcriptase polymerase chain reaction (RT-PCR) analysis, 5 PMCAs and 2 NCXs were ubiquitously expressed in various tissues including gills. Triple fluorescence in situ hybridization and immunocytochemistry showed the colocalization of *zecac*, *zpmca2*, and *zncx1b *mRNAs in a portion of gill MR cells (using Na^+^-K^+^-ATPase as the marker), implying a subset of ionocytes specifically responsible for the transepithelial Ca^2+ ^uptake in zebrafish gills. The gene expressions in gills of high- or low-Ca^2+^-acclimated zebrafish by quantitative real-time PCR analysis showed that *zecac *was the only gene regulated in response to environmental Ca^2+ ^levels, while *zpmcas *and *zncxs *remained steady.

**Conclusion:**

The present study provides molecular evidence for the specific isoforms of Ca^2+ ^transporters, zECaC, zPMCA2, and zNCX1b, supporting the current Ca^2+ ^uptake model, in which ECaC may play a role as the major regulatory target for this mechanism during environmental challenge.

## Background

Ca^2+ ^is an essential element for almost all organisms and plays comprehensive regulatory roles in eukaryotic cells; therefore, Ca^2+ ^must be maintained within a narrow concentration range in organisms (cellular free [Ca^2+^]: 100 nM; plasma total [Ca^2+^]: 2~3 mM). In terrestrial vertebrates, the whole-body Ca^2+ ^balance is mainly achieved by intestinal absorption and kidney reabsorption. Ca^2+ ^uptake mechanisms in the mammalian kidney have been most extensively studied, while fish gills, a specialized organ in non-mammalian vertebrates, is another model for studying Ca^2+ ^uptake mechanisms [1]. In fish, gills are the main site (> 97% of the whole body) of Ca^2+ ^uptake from the aquatic environment to maintain the Ca^2+ ^balance [2], and skin takes the place of gills during early developmental stages when the gills are not yet developed and functioning [3-5]. Compared with terrestrial vertebrates, Ca^2+ ^regulation mechanisms in aquatic animals are probably more complicated and challenging because of the dramatic fluctuations in ambient Ca^2+ ^concentrations which can occur (seawater, around 10 mM; fresh water, 0.01~3 mM).

According to the current model in mammals, active and transcellular Ca^2+ ^transport is carried out as a 3-step process [1]. Following entry of Ca^2+ ^through apical epithelial Ca^2+ ^channels (ECaC, TRPV5, and/or TRPV6), Ca^2+ ^is bound to calbindins that facilitate diffusion to the basolateral membrane, and then it is extruded via the plasma membrane Ca^2+^-ATPase (PMCA) and/or Na^+^/Ca^2+ ^exchanger (NCX). In this way, net transepithelial Ca^2+ ^absorption from the luminal compartment (or environment) to the plasma is accomplished. A model of the capability of physiological regulation has also been similarly proposed in fish gills [6]. However, only very little molecular evidence is currently available to support this model in fish gills, which is a specialized organ for Ca^2+ ^uptake. The *ecac *gene has recently been cloned and sequenced in fugu, zebrafish, and trout [4,7,8]. In zebrafish embryos, low-Ca^2+ ^fresh water causes upregulation of the whole-body Ca^2+ ^influx and zECaC expression in mitochondrion-rich (MR) cells of both gills and skin, providing molecular evidence for the role of ECaC in fish Ca^2+ ^uptake [4]. On the other hand, some biochemical and physiological studies have investigated the possibility of PMCA and NCX's involvement in fish gill Ca^2+ ^uptake [9,10]. Nevertheless, no convincing evidence has been presented demonstrating the existence and involvement of PMCA and NCX in fish Ca^2+ ^uptake mechanisms.

Three NCX genes (SLC8A1, SLC8A2, and SLC8A3) and 4 PMCA genes (ATP2B1, ATP2B2, ATP2B3, and ATP2B4) have been identified in mammals so far. The fundamental work of PMCA is the highly regulated active extrusion of Ca^2+ ^from cells for maintaining a gradient across the plasma membrane [11]. PMCA belongs to the P-type primary ion transport ATPase superfamily with 10 transmembrane domains and 1 calmodulin-binding site. At least 20 alternative splicing variants of PMCA have been found in mammals [12]. In mammals, PMCA1 and PMCA4 are ubiquitously expressed, whereas PMCA2 and PMCA3 are more tissue specific; it has been suggested that PMCA1 and PMCA4 are housekeeping isoforms involved in the maintenance of cellular Ca^2+ ^homeostasis [13]. However, no molecular data about PMCAs are available in fish. On the other hand, at least 32 alternatively spliced isoforms of NCX1 gene products have been identified [14]. Knock-out of NCX1 in mice is lethal due to cardiac defects before birth [15], while many physiological studies have indicated that vitamin D_3 _modulates Ca^2+ ^balance by upregulating NCX1 expression [16]. Recently, NCX1-3 and NCX4, another putative prototype of NCX, have been reported in fish, indicating the existence of NCXs in non-mammals as well [17,18]. However, there is no molecular physiological evidence to support the involvement of NCX in fish Ca^2+ ^uptake mechanisms.

Because of a theoretical process of whole-genome duplication (WGD) from ancestor vertebrates [19,20], the numbers of gene isoforms found in teleosts are usually more than those found in mammals and tetrapods. While different isoforms coded from distinct genomic loci tend to have tissue-specific expressions, there are some cases of isoforms being differentially regulated upon facing environmental changes (reviewed by Schulte [21]. Isoforms also serve compensatory functions; upregulation of TRPV6 was found in the kidneys of TRPV5-deficient mice [22]. In biochemical studies on fish, the gill activities of PMCA and NCX, which are altered based upon physiological needs and environmental changes, have been proposed as being associated with transepithelial Ca^2+ ^transport [9,23]. However, nothing is known about which specific isoforms are responsible for Ca^2+ ^uptake mechanisms in teleost gills. Therefore, using a functional genomic approach to investigate all isoforms of PMCA and NCX in a species may provide both physiological and genomic insights into this issue.

In the present study, a strategy of a whole-genome survey was used to uncover any unidentified PMCA and NCX isoforms in zebrafish, and then these candidates were refined with the EST database and cloned. Six isoforms of PMCAs (PMCA1a, PMCA1b, PMCA2, PMCA3a, PMCA3b, and PMCA4) and 7 isoforms of NCXs (NCX1a, NCX1b, NCX2a, NCX2b, NCX3, NCX4a, and NCX4b) were identified. Moreover, we report that some duplication of PMCA and NCX occurred, and the tissue distributions and expression patterns of these isoforms were analyzed by reverse-transcriptase polymerase chain reaction (RT-PCR) and triple fluorescence labeling, respectively. The gene expressions in gills of zebrafish acclimated to high- or low-Ca^2+ ^environments were also examined by quantitative real-time PCR analysis. The present study provides molecular evidence to support the current Ca^2+ ^uptake model in fish gill cells [24].

## Results

### Molecular cloning and bioinformatics analysis of *pmca *and *ncx*

**Figure 1:**
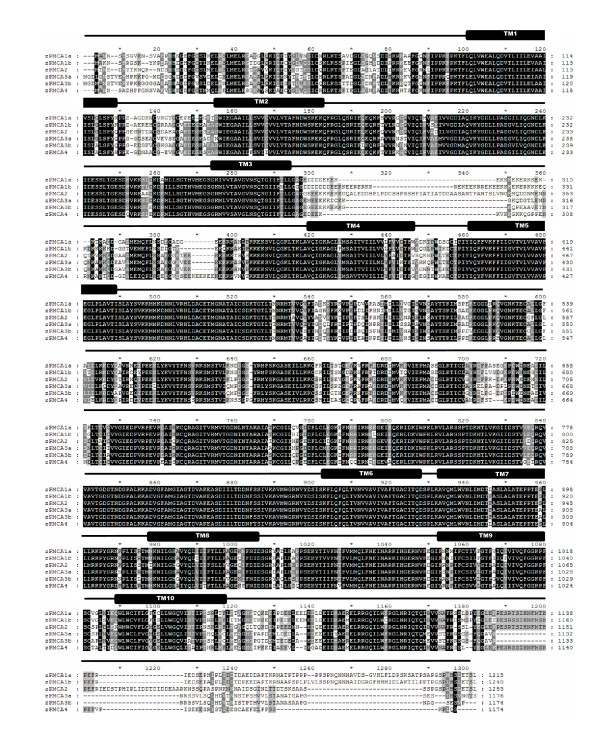
Alignment of the deduced amino acid sequences of zebrafish plasma membrane Ca^2+^-ATPases (PMCAs). Residues in black and gray regions indicate identical and similar residues between isoforms, respectively. TM1-10, the postulated transmembrane domains.

**Figure 2:**
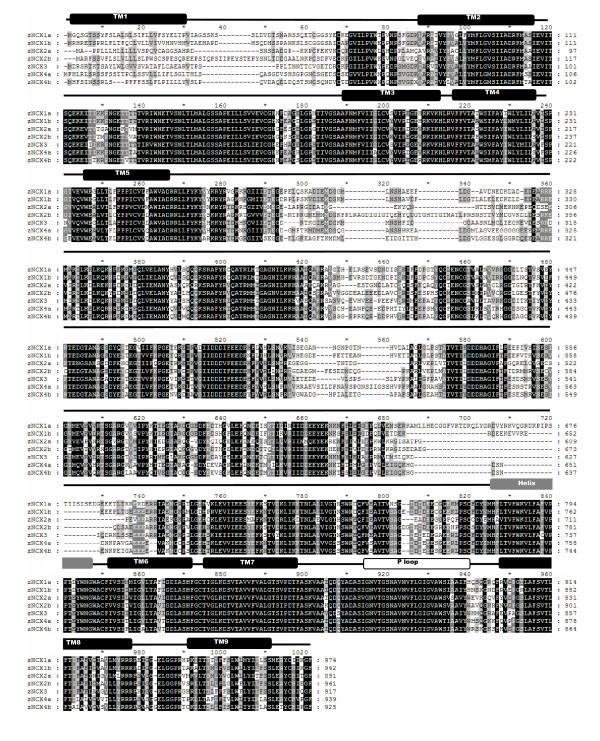
Alignment of the deduced amino acid sequences of the zebrafish Na^+^/Ca^2+ ^exchangers (NCXs). Residues in black and gray regions indicate identical and similar residues between isoforms, respectively. The intracellular helixes (Helix, gray box) and the "GIG" P loops (P loop, open box) of NCX are also indicated. TM1-9, the postulated transmembrane domains.

**Figure 3:**
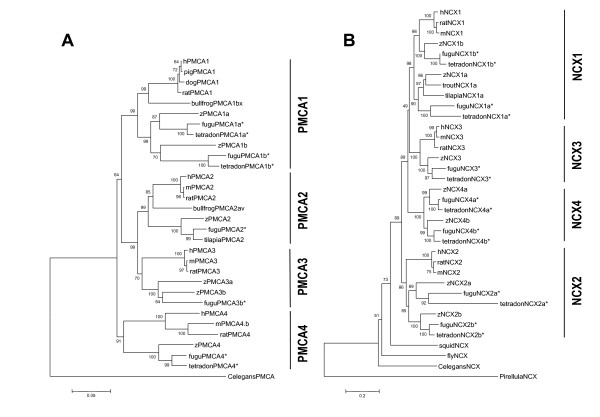
Phylogenetic analysis of plasma membrane Ca^2+^-ATPase (PMCA) and Na^+^/Ca^2+ ^exchanger (NCX) amino acid sequences. Consensus trees were generated using a Neighbor-joining (NJ) method with 1000-time bootstraps. The GenBank accession numbers of the sequences used are as follows: human PMCA 1 (hPMCA1), P20020; human PMCA 2 (hPMCA2), Q01814; human PMCA 3 (hPMCA3), Q16720; human PMCA 4 (hPMCA4), P23634; Norway rat PMCA 1 (ratPMCA1), P11505; Norway rat PMCA 2 (ratPMCA2), P11506; Norway rat PMCA 3 (ratPMCA3), Q64568; Norway rat PMCA 4 (ratPMCA4), Q64542; mouse PMCA 2 (mPMCA2), NP_033853; mouse PMCA 3 (mPMCA3), NP_796210; mouse PMCA 4.b (mPMCA4.b), AY560895; dog PMCA 1 (dogPMCA1), NP_777121; pig PMCA 1 (pigPMCA1), P23220; bullfrog PMCA 1bx (bullfrogPMCA1bx), AAK11272; bullfrog PMCA 2av (bullfrogPMCA2av), AAK11273; *Caenorhabditis elegans *PMCA (CelegansPMCA), CAA09303;*Oreochromis mossambicus *PMCA 2 (tilapiaPMCA2), AAK15034; *Tetraodon nigroviridis *unnamed protein product (tetradonPMCA1a), CAF95990; *T. nigroviridis *unnamed protein product (tetradonPMCA1b), CAG08760; *T. nigroviridis *unnamed protein product (tetradonPMCA4), CAF90203; human NCX 1 (hNCX1), P32418; human NCX 2 (hNCX2), Q9UPR5; human NCX 3 (hNCX3), NP_150287; mouse NCX 1 (mouseNCX1), P70414; mouse NCX 2 (mouseNCX2), NP_683748; mouse NCX 3 (mouseNCX3), NP_536688; Norway rat NCX 1 (ratNCX1), Q01728; Norway rat NCX 2 (ratNCX2), NP_511174; Norway rat NCX 3 (ratNCX3), NP_511175; *T. nigroviridis *unnamed protein product (tetraodonNCX1), CAG00274; *T. nigroviridis *unnamed protein product (tetraodonNCX2a), CAF95011; *T. nigroviridis *unnamed protein product (tetraodonNCX2b), CAG06357; *T. nigroviridis *unnamed protein product (tetraodonNCX3), CAG13245; *T. nigroviridis *unnamed protein product (tetraodonNCX4a), CAG05743; *T. nigroviridis *unnamed protein product (tetraodonNCX4b), CAG01582; *Ore. mossambicus *NCX1a (tilapiaNCX1a), AAP37041; *Oncorhynchus mykiss *NCX 1a (troutNCX1a) AAF06363; *Rhodopirellula baltica *NCX (PirellulaNCX), NP_868143; *C. elegans *NCX (CelegansNCX), CAA04574; *Loligo opalescens *NCX (squidNCX), AAB52920; and *Drosophila melanogaster *NCX (flyNCX), NP_524423. The un-annotated proteins of *Takifugu rubripes *obtained from Ensembl browser with the Ensembl gene ID are as follows: *Takifugu *PMCA 3b (fuguPMCA3b), NEWSINFRUG00000123527; *Takifugu *PMCA 2 (fuguPMCA2), NEWSINFRUG00000164849; *Takifugu *PMCA 1a (fuguPMCA1a), NEWSINFRUG00000133763; *Takifugu *PMCA 4 (fuguPMCA4), NEWSINFRUG00000161238; *Takifugu *PMCA 1b (fuguPMCA1b), NEWSINFRUG00000135851; *Takifugu *NCX 4b (fuguNCX4b), NEWSINFRUG00000165032; *Takifugu *NCX 4a (fuguNCX4a), NEWSINFRUG00000131148; *Takifugu *NCX 1a (fuguNCX1a), NEWSINFRUG00000136894; *Takifugu *NCX 1b (fuguNCX1b), NEWSINFRUG00000145936; *Takifugu *NCX 3 (fuguNCX3), NEWSINFRUG00000135815; *Takifugu *NCX 2b (fuguNCX2b), NEWSINFRUG00000136441; and *Takifugu *NCX 2a (fuguNCX2a), NEWSINFRUG00000160221. An asterisk (*) indicates a hypothetical protein predicted from other studies.

In this whole-genome survey, 6 distinct PMCAs (Figure 1) and 7 distinct NCXs (Figure 2) were found in zebrafish. According to the phylogenetic analysis, these transporters were annotated as zPMCA1a, zPMCA1b, zPMCA2, zPMCA3a, zPMCA3b, zPMCA4 (Figure 3a, and in standard nomenclature ATP2B1a, ATP2B1b, ATP2B2, ATP2B3a, ATP2B3b, and ATP2B4, respectively) and zNCX1a, zNCX1b, zNCX2a, zNCX2b, zNCX3, zNCX4a, and zNCX4b (Figure 3b, in standard nomenclature SLC8a1a, SLC8a1b, SLC8a2a, SLC8a2b, SLC8a3, SLC8a4a, and SLC8a4b, respectively). zNCX1a and zNCX1b were previously described and named zNCX1h and zNCX1n, respectively [25], and zNCX2a, zNCX3, zNCX4a, and zNCX4b have also been published [17,18]. However, zNCX2b and the other zPMCAs are first described in this study.

**Figure 4:**
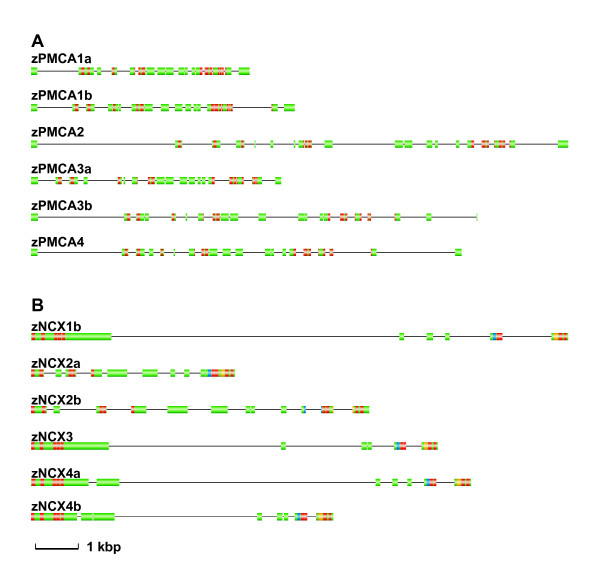
Gene structures of plasma membrane Ca^2+^-ATPases (PMCAs) and Na^+^/Ca^2+ ^exchangers (NCXs). Exons are showed in gradient bars separated by introns that are drawn in lines. The scale bar for the length of exon is shown, and introns are shown as 1/10 the size of exons. Red-filled region, the predicted transmembrane domain; blue-filled region, the intracellular helixes of NCX; orange-filled exon region, the GIG P loops of NCX.

Based on the topological structures of PMCA and NCX summarized in mammals [12,26], zebrafish PMCAs and NCXs share similar patterns as mammals at the deduced amino acid sequences. According to the hydropathy analysis, 10 putative transmembrane domains were predicted for zPMCA (Figures 1, 4a), whereas NCX has 11 hydrophobic segments and 9 putative transmembrane domains (Figures 2, 4b) due to an intracellular helix front transmembrane domain 6 and a P loop-like structure of hydrophobic segment 8, which contained the amino acid sequence "GIG" [26]. PMCA and sarco-endoplasmic reticulum calcium ATPase (SERCA), the closest kin to PMCA, share a similar topological structure: hydrophobic segments span the membrane 10 times, and 3 intracellular loops consist of Ca^2+ ^binding and transporting, and calmodulin-regulating functions [12,27]; the zPMCAs were also found to have 3 cytoplasmic domains 1 each between TM2 and TM3, TM4 and TM5, and TM10 and the C-terminal (Figure 1). As for NCX, a large intracellular loop for ion translocation [28,29] was located between TM5 and TM6 (Figure 2). There were 20 alternative splicing isoforms of zPMCAs found when cloning was conducted, while 9 alternative splicing isoforms were found in zNCXs (data not shown). The hot spots of alternative splicing regions in the zPMCAs are at the 5' untranslated regions (UTRs), the fragments around the 300^th ^amino acid including from 1 to 3 small exons (less than 100 bp each in those cases), and 3' amino acid tails that usually stretch to the 3' UTR and alter the location of stop codons. On the other hand, a region of alternative splicing sites was found in zNCX1b and zNCX3 around the 610^th ^amino acid, which is a part of the intracellular loop.

zPMCAs share 63%~86% identities with each other, while zNCXs exhibit higher variety among isoforms (i.e., 36%~76% identity). According to the phylogenetic analysis, zebrafish and 2 pufferfish species so far all have 6 PMCA and 7 NCX isoforms (Figure 3a,b). Compared with the isoforms annotated in tetrapods, duplication from the ancestor ray-finned fish occurred in PMCA1, PMCA3, NCX1, NCX2, and NCX4, according to the latest version of the genomic database utilized. Moreover, in the karyotype, zPMCAs and zNCXs are located on different chromosomes (see additional file 1), suggesting that these paralogous groups may have originated from genome duplication but not from tandem duplication. Relationships among these pairs of duplicates were evident not only in the phylogenetic tree, but also in their gene structures (Figure 4). All teleost PMCAs and NCXs examined in this study showed an outgroup topology to their orthologues of mammals (Figure 3a,b), and the paralogous teleosts formed an inner-group topology. The 2 prototypes of NCXs, NCX4a and NCX4b, found in teleosts were most closely related to NCX1 and NCX3 in both phylogenetic trees (Figure 3) and gene structures (Figure 4b). In gene structures, all of them had a relatively large first exon, even though some small introns were found in the split exons in zNCX4s (Figure 4b).

### mRNA expressions of *pmca *and *ncx *in various tissues

**Figure 5:**
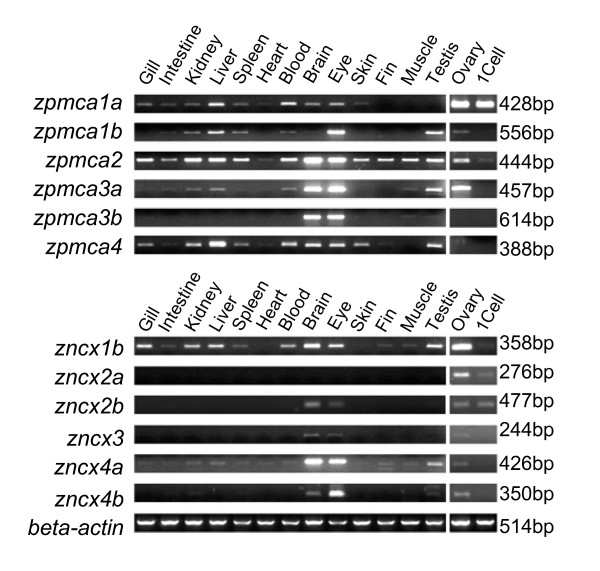
Expression patterns of zebrafish plasma membrane Ca^2+^-ATPases (PMCAs) and Na^+^/Ca^2+ ^exchangers (NCXs) in various tissues by RT-PCR analysis. The zebrafish beta-actin was used as internal control to evaluate the relative amounts of cDNAs. The expected amplicon sizes are shown on the right. 1Cell, cDNA from the 1-cell stage of zebrafish embryos.

RT-PCR analysis revealed that *zpmca2 *and *zpmca4 *were ubiquitously expressed in various tissues compared with *zpmca1a*, *zpmca1b*, *zpmca3a*, and *zpmca3b *(Figure 5). All *zpmcas *were abundant in the brain and eye except *zpmca1b*, which was mainly expressed in the eye. These findings agree with the Ca^2+ ^homeostasis function of PMCAs found in the mammalian neuron system [12]. *zncx1b *and *zncx4a *were also ubiquitously expressed, while *zncx2b *and *zncx3 *were only detected in the brain and eyes. *zpmca1a*, *zpmca1b*, *zpmca2*, *zpmca3a*, and all the *zncxs *were expressed in ovary and 1-cell embryo (Figure 5), suggesting that these genes may regulate intracellular Ca^2+ ^homeostasis at earlier stages. *zpmca1a*, *zpmca2*, *zpmca4*, *zncx1*, and *zncx4a *were expressed in gills (Figure 5).

### Effects of environmental Ca^2+ ^levels on *ecac*, *pmca*, and *ncx *mRNA expressions

**Figure 6:**
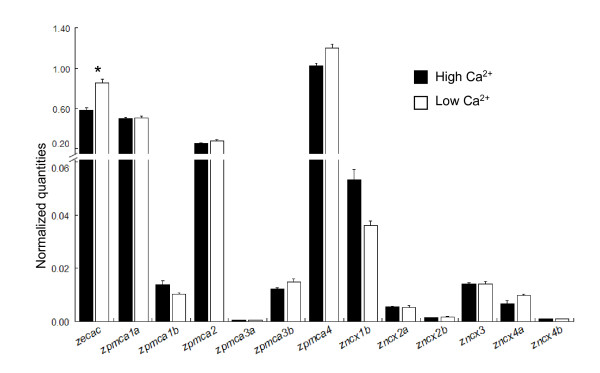
Quantitative real-time PCR analysis of plasma membrane Ca^2+^-ATPases (PMCAs) and Na^+^/Ca^2+ ^exchangers (NCXs). HCa (filled bar), cDNA from the gill of high-Ca^2+^-acclimated zebrafish; LCa (opened bar), cDNA from the gill of low-Ca^2+^-acclimated zebrafish. An asterisk (*) indicates a significant difference between the 2 groups (Student's *t*-test, *p *< 0.05).

According to the quantitative real time-PCR analysis, low-Ca^2+ ^FW-acclimated zebrafish expressed significantly higher levels of *zecac *in gills than did high-Ca^2+ ^FW-acclimated fish (Figure 6). However, no significant difference was found between the 2 groups in the expressions of any of the *zpmcas *and *zncxs *(Figure 6). Consistent with the results of the tissue scans shown in Figure 5, *zpmca4*, *zpmca1a*, and *zpmca2 *exhibited more than 20-fold higher expression levels than did the other *zpmca *isoforms in gills, whereas *zncx1b *was found to have an approximately 5-times stronger signal compared with the other *zncxs *(Figure 6).

### Colocalization of *ecac*, *pmca*, and *ncx *mRNAs and Na^+^-K^+^-ATPase

Fluorescence *in situ *hybridization and immunocytochemistry were used to determine the isoforms of *zpmca *and *zncx *that were specifically expressed in gill ionocytes. Specific mRNA probes of all of the *zpmca *and *zncx *isoforms were used to conduct the *in situ *hybridizations for whole-mount embryos and adult gills. Among the isoforms, only *zpmca2 *and *zncx1b *mRNA signals were found in specific groups of cells in embryonic skin and adult gills (data not shown).

**Figure 7:**
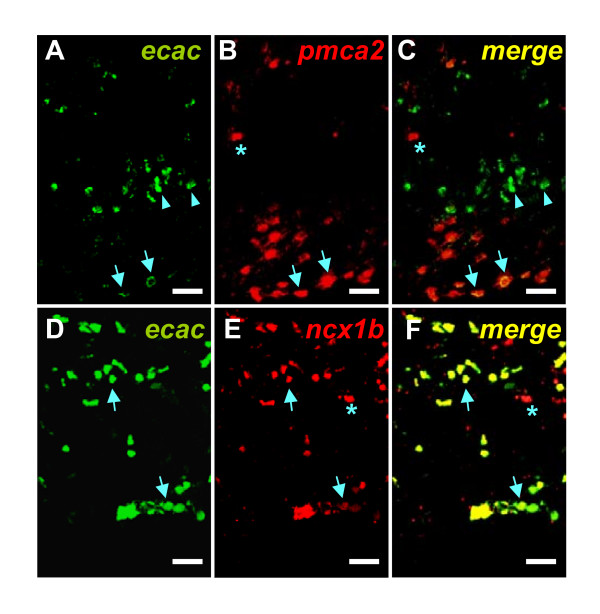
Double fluorescence *in situ *hybridizations of *zecac*/*zpmca2 *(A-C) and *zecac*/*zncx1b *(D-F) in frozen sections of zebrafish gills. A and D, z*ecac *mRNA; B, *zpmca2 *mRNA; C, merged image of A and B; E, *zncx1b *mRNA; F, merged image of D and E. Arrow indicates the colocalization of *zecac*/*zpmca2 *(A-C) and *zecac*/*zncx1 *(D-F), respectively. An asterisk (*) indicates the *zpmca2 *signal without *zecac *colocalization (A and B) and the *zncx1b *signal without *zecac *colocalization (D and E). Scale bar = 20 μm.

**Figure 8:**
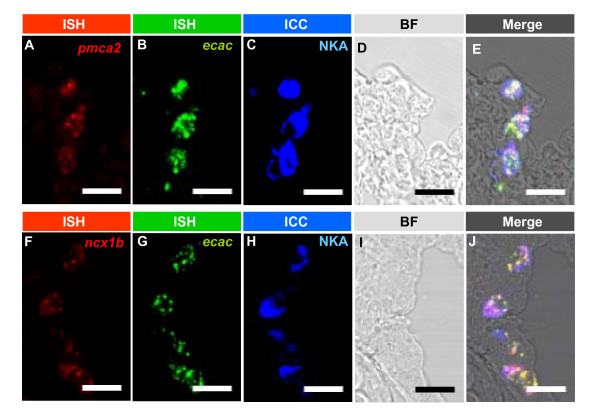
Triple fluorescence *in situ *hybridizations (ISH) and immunocytochemistry (ICC) for *zecac *mRNA/*zpmca2 *mRNA/Na^+^-K^+^-ATPase (A-E) and *zecac *mRNA/*zncx1b *mRNA/Na^+^-K^+^-ATPase (F-J) in frozen sections of zebrafish gills. A-E are the same section, and F-J are another section. A, *zpmca2 *mRNA; B and G, *zecac *mRNA; C and H, Na^+^-K^+^-ATPase (NKA); D and I, bright field (BF); E, merged image of A-D; J, merged image of F-I. Colocalization of the 3 transporter mRNAs and protein was found respectively in E and J. Scale bar = 20 μm.

Subsequently, double-fluorescence *in situ *hybridizations were used for *zecac *and z*pmca2 *(Figure 7A–C), and *zecac *and z*ncx1b *(Figure 7D–F), respectively. More gill cells expressed z*pmca2 *and z*ncx1b *mRNAs than z*ecac *mRNA (Figure 7A–F). Notably, z*pmca2 *and z*ncx1b *were also abundantly expressed in gill lamellae, where fewer MR cells are usually found [24]. *zecac *and z*pmca2 *mRNAs were colocalized only in a portion of gill cells, but were not colocalized all of the time. Around half of the *zecac*-positive cells co-expressed *zpmca2 *signals (51.7 +/- 3.50%, *n *= 3), while only 40.3 +/- 1.35% (*n *= 3) of *zpmca2*-positive cells showed *zecac *signals (Figures 7A–C, 8). Similar results were found in the case of double labeling of *zecac *and *zncx1b *(Figures 7D–F, 8). The proportion of z*ncx1b-*positive cells that co-expressed *zecac *was 77.1 +/- 1.35% (*n *= 3), while *zncx1b *signals could be detected in all *zecac*-positive cells (Figures 7D–F, 8).

The subsequent triple-labeling experiments further demonstrated the colocalization of *zecac *mRNA/*zpmca2 *mRNA/Na^+^-K^+^-ATPase (Figure 8A–E), and *zecac *mRNA/*zncx1b *mRNA/Na^+^-K^+^-ATPase (Figure 8F–J), respectively, in specific groups of gill ionocytes. Taken all together, only a portion of gill ionocytes co-expressed the mRNA of *zecac*, *zpmca2*, and *zncx1b*, and Na^+^-K^+^-ATPase.

## Discussion

So far, 4 PMCA genes have been identified in mammals, and PMCA1 and PMCA4 were suggested to be housekeeping isoforms. Mammalian PMCA2 was found to be expressed in the hair bundle of hair cells of the inner ear [30], suggesting that it is responsible for endolymph Ca^2+ ^homeostasis [11]. A null mutation of PMCA2 caused balance and hearing defects in mice [31]. On the other hand, mammalian PMCA1 was deemed a major Ca^2+ ^extrusion transporter for Ca^2+ ^absorption in intestine and kidney [32-34]. Our data demonstrated opposite phenomena in fish PMCAs. *zpmca2 *was expressed with a housekeeping pattern (Figure 5), while *zpmca1a *showed a hair cell expression pattern during embryonic stages (data not shown). This functional substitution of paralogues found in Ca^2+ ^transporters is not a unique example. In zebrafish, the expression profiles suggest the hypothesis that zebrafish hoxB1a and hoxB1b are functional equivalents of mouse Hoxb1 and Hoxa1, respectively. On the contrary, the zebrafish orthologue of mouse Hoxa1, zebrafish hoxA1a, is not expressed in a similar pattern [20]. Further examination of the promoter regions of these genes may provide insights into this phenomenon. As far as our data, zPMCA2 was proposed to participate in the transcellular Ca^2+ ^uptake pathway in fish gills. NCX1a and NCX1b from teleosts including zebrafish and pufferfish were not clustered within an inner group on the phylogenetic tree (Figure 3). Further collection is necessary to enlarge the sequence pool, which may allow us to reconstruct the phylogenetic tree or to rename members of the gene family. In the present study, zNCX1b was demonstrated to be responsible for the Ca^2+ ^extrusion mechanism in fish gill cells, while the paralogue, zNCX1a, was proven to be a heart-specific isoform that causes cardiac fibrillations in zNCX1a mutants [25]. The combined functions from both zNCX1s are equivalent to those of mammalian NCX1; however, investigations of the direct function of NCX1 in Ca^2+ ^absorption were not possible due to fatal heart failure in knockout mice. Therefore, teleosts may serve as a good model to examine the role of NCX in Ca^2+ ^uptake without resulting heart failure.

As described above, Flik et al. [6] proposed a model of a fish gill Ca^2+^uptake mechanism, which is similar to that for Ca^2+ ^reabsorption in mammalian kidneys [1]. There has been no convincing or comprehensive molecular evidence to support the existence of the 3 major transporters, ECaC, PMCA, and NCX, in fish gill ionocytes until the present study. Pan et al. [4] for the first time demonstrated the expression of *zecac *in a subset of MR cells in zebrafish skin/gills and a correlation between *zecac *expression and *in vivo *Ca^2+ ^uptake function. Subsequently in rainbow trout, Perry and colleagues found the expression of ECaC mRNA and/or protein in both pavement and MR cells in rainbow trout [8,35], which is inconsistent with the current view that MR cells are the predominant site for branchial Ca^2+ ^uptake [36], and the authors also reported that hypercapnia, implantation with cortisol, and infusion with CaCl_2 _affected ECaC expression in MR cells and/or pavement cells [35]. In a recent study on isolated trout gill cells, PNA^+ ^MR cells showed an over 3-fold higher ^45^Ca^2+ ^uptake capacity compared to either PNA^- ^MR or pavement cells [37]. The subset of gill MR cells and/or pavement cells that expressed ECaC is not necessarily involved in transepithelial Ca^2+ ^uptake as Hwang and Lee [24] claimed, because those previous studies lacked molecular and physiological data to demonstrate the transport pathway across the basolateral membrane. The present triple *in situ *hybridization and immunocytochemical experiments demonstrate the co-expressions of *zecac*, *zpmca2*, and *zncx1b *in a specific group of gill MR cells, providing comprehensive molecular evidence for the involvement of these transporters in a fish gill Ca^2+ ^uptake mechanism.

Double *in situ *hybridization and immunocytochemistry experiments indicated that about 90% of gill MR cells expressed *zecac *mRNA, although this may have been underestimated because the antigenicity of Na^+^-K^+^-ATPase (the marker for MR cells) is attenuated during the high-temperature treatments used for *in situ *hybridization. All *zecac*-expressing cells co-expressed *zncx1b*, and about half of the *zecac*-expressing cells co-expressed *zpmca2*, indicating that about 50% of *zecac*-expressing cells co-expressed both *zncx1b *and *zpmca2*. Taking all these findings into account, only about 45% of the entire population of MR cells co-express *zecac*, *zpmca2*, and *zncx1b*, and this subset of ionocytes may specifically carry out the function of transepithelial Ca^2+ ^uptake in zebrafish gills. It was noted that some gill cells express only 1 or 2 (never all) of the 3 major Ca^2+ ^transporters. These cells may be in the process of terminal differentiation as Hsiao et al. [38] reported in skin ionocytes in zebrafish embryos. Alternatively, the Ca^2+ ^transporter(s) expressed in those cells may be involved in intracellular Ca^2+ ^homeostasis or other cellular events [4,39,40]. This remains to be confirmed in future studies.

ECaC is a vitamin D-sensitive transporter in mammals [22], and the expression of PMCA1 was also found to be stimulated by 1,25(OH)_2_D_3 _in mammals and chicken [34,41,42]. 1,25(OH)_2_-vitamin D_3 _was shown to have a hypercalcemic effect on both freshwater and saltwater teleosts [6,43], whereas stanniocalcin, a predominant hypocalcemic hormone produced by Stannius bodies, was found to show opposite effect on fish [44]. Stanniocalcin did not show a significant effect on basolateral Ca^2+ ^transport [45], but quickly reduced the permeability of Ca^2+ ^at the apical membrane [6,46]. It has been proposed that ECaC is the rate-limiting step and the gatekeeper channel for active Ca^2+ ^transport [47]. The present study provides further molecular physiological evidence to support this notion. The present study for the first time demonstrates that acclimation to a low-Ca^2+ ^environment, which would stimulate Ca^2+ ^uptake capacity in fish [3,48,49], caused upregulation of mRNA expression of zECaC but not zPMCA2 or zNCX1b, implying that the steady-state expressions of zPMCA2 and zNCX1b may fulfill the requirement for the transepithelial transport machinery under all situations. Moreover, this study provides molecular evidence to support a previous biochemical study by Flik et al. [50], who found that the basolateral Ca^2+ ^extrusion mechanisms by PMCA and NCX is far below their maximum capacity in fish gills based on the kinetic properties of the 2 enzymes.

## Conclusion

The major findings of the present study were that (1) 6 PMCAs and 7 NCXs were identified from zebrafish tissues; (2) differential expressions of these genes were found in various tissues including gills; (3) acclimation to low-Ca^2+ ^environment stimulated the mRNA expression of *ecac *in gills, but not those of *pmcas *or *ncxs*; and (4) only a portion of gill MR cells co-expressed *zecac, zpmca2*, and *zncx1b *mRNAs.

## Methods

### Animals and embryos

Zebrafish (*Danio rerio*) brood stocks at the Institute of Cellular and Organismic Biology, Academia Sinica were kept in fresh water (local tap water, FW) at 28.5°C under a 14-h:10-h light: dark photoperiod. Embryos were collected within 30 min after fertilization and incubated in a Petri dish until the desired developmental stages. For whole-mount *in situ *hybridization, PTU (1-phenyl 2-thiourea) at a final concentration of 0.003% was added to prevent melanogenesis. Fish were anesthetized with buffered MS222 before sampling following the guidelines of the Academia Sinica Institutional Animal Care and Utilization Committee (approval no.: RFiZOOHP2006086).

### Acclimation experiment

Following a previous process in our laboratory [49], high-Ca^2+ ^(2.00 mM, control) and low-Ca^2+ ^(0.02 mM) artificial fresh waters were prepared with double-deionized water (Milli-RO60, Millipore, Billerica, MA, USA) supplemented with adequate CaSO_4_·2H_2_O, MgSO_4_·7H_2_O, NaCl, K_2_HPO_4_, and KH_2_PO_4_. Nominal Ca^2+ ^concentrations of the high- and low-Ca^2+ ^media were 2.00 and 0.02 mM, respectively, but the other ion concentrations of the media were the same ([Na^+^], 0.5 mM; [Mg^2+^], 0.16 mM; and [K^+^], 0.3 mM) as those in the local tap water. Variations in the ion concentrations were maintained within 10% of the predicted values by examination with an atomic absorption spectrophotometer (Hitachi Z-8000, Tokyo, Japan). Zebrafish were transferred to high- and low-Ca^2+ ^media, respectively, for 2 wk. At the end of acclimation, fish gills were sampled for quantitative real-time PCR analysis.

### Molecular cloning and sequences analysis

The peptide sequences from other species (teleost had a higher priority) were used to BLAST the genome databases (of NCBI and Ensembl) for zebrafish, pufferfish (*Fugu rubripes*), and tetradon (*Tetraodon nigroviridis*). The putative full-length or partial open reading frames of PMCAs and NCXs obtained were confirmed using the EST database, and/or were used to design primers for cloning and the RT-PCR analysis. PCR products thus obtained were subcloned into a pGEM-T Easy vector (Promega, Madison, WI, USA), and the nucleotide sequences were determined with an ABI 377 sequencer (Applied Biosystems, Warrington, UK). Sequence analysis was conducted with a BLASTx program (NCBI). The specific primers of 5' and 3'-rapid amplification of cDNA ends (RACE) were designed from the partial sequences obtained from the PCR with degenerate primers. The RACE PCR program followed a commercial protocol (Clontech, Mountain View, CA, USA), and the RACE PCR products were also subcloned into pGEM-T Easy vectors and sequenced. The full-length deduced amino-acid sequences were aligned with ClustalX and then phylogenetically analyzed with Mega3.1 [51]. For the proteomic structure analysis, the hydropathy plot and transmembranes were predicted with EMBOSS (the European Molecular Biology Open Software Suite, version 2.8.0 [52] algorithm according to Persson and Argos [53].

### RT-PCR analysis

An appropriate amount of zebrafish adult tissues was collected for the total RNA preparation with TRIZol reagent (Invitrogen, Carlsbad, CA, USA). The amount and quality of total RNA were determined by measuring the absorbances at 260 and 280 nm with a spectrophotometer (Hitachi U-2000) and RNA denatured gels. For cDNA synthesis, 5 μg of total RNA was reverse-transcribed in a final volume of 20 μL containing 0.5 mM dNTPs, 2.5 μM oligo (dT)_18_, 5 mM dithiothreitol, and 200 units superscript reverse transcriptase III (Invitrogen) for 1.5 h at 42°C, followed by a 15-min incubation at 70°C. For the PCR amplification, 1 μL cDNA was used as a template in a 25-μL final reaction volume containing 0.25 mM dNTP, 1.25 units Gen-Taq polymerase (Genemark, Taipei, Taiwan), and 0.2 μM of each primer. The primer sets for the PCR of tissue scans were zPMCA1a (428-bp fragment) forward 5'-AGACAGGGTGGATAGAAGGT-3', reverse 5'-CCCCAATAATGTGAAGATGA-3'; zPMCA1b (556-bp fragment), forward 5'-AGCCCCTTATTTCCCGCAC-3', reverse 5'-GCCCCCTTCTCAGCTCCATT-3'; zPMCA2 (444-bp fragment) forward 5'-GGGATCGGGATGAGATGGTA-3', reverse 5'-GCGTGTGTTTGTCTGTGGGT-3'; zPMCA3a (457-bp fragment) forward 5'-TTGCTGGTACAGATGTGGC-3', reverse 5'-GGAGGAGAGTGAAGCGGAG-3'; zPMCA3b (614-bp fragment) forward 5'-GGTCTGCTGGGTTTCCTACT-3', reverse 5'-TCCTGCTCAATCTCTCCTTT-3'; zPMCA4 (388-bp fragment) forward 5'-GTCAAGGCTGTGATGTGGGC-3', reverse 5'-TGGTTGGGAGTGGAGAAGGG-3'; zNCX1b (358-bp fragment) forward 5'-AGAGACGAGGAGAAGGAGGT-3', reverse 5'-GCACGAAAGCAAAGAGAACT-3'; zNCX2a (276-bp fragment) forward 5'-TGATGAAGAAGGAGGTGAAC-3', reverse 5'-CTTGCGAATGTGTCTGGTAT-3'; zNCX2b (477-bp fragment) forward 5'-GCAGCCGCATTTCCTTCG-3', reverse 5'-CGCCAGAGTTTCGGACCAC-3'; zNCX3 (244-bp fragment) forward 5'-GAGGAAGCAAGAAGAATAGC-3', reverse 5'-AGTCAAAACAAGATGGCAGA-3'; zNCX4a (462-bp fragment) forward 5'-GGAAGAGAGCGGAGAAGAGC-3', reverse 5'- ATGGTGAAGAGGGTGACGGA-3'; and zNCX4b (350-bp fragment) forward 5'-ATGCAGCAGCAGAAGAGC-3', reverse 5'-ACATTCTCGCCAGGTTTG-3'. A 514-bp fragment of zebrafish beta-actin was used as an internal control to evaluate the relative amounts of complementary DNAs (cDNAs), and the design of the primer pairs followed Hsiao *et al*. [54]. The amplicons were all sequenced to make sure that the PCR products were the desired gene fragments.

### Fluorescence double *in situ *hybridization

Fragments of the target genes obtained by PCR were inserted into pGEM-T Eeasy vectors. Digoxigenin- (Dig) (Roche, Penzberg, Germany) or dinitrophenol (DNP)-labeled (Perkin-Elmer, Boston, MA, USA) RNA probes were synthesized by *in vitro *transcription with T7 and SP6 RNA polymerase (Takara, Shiga, Japan). The qualities of the probes were examined using RNA gels, and the concentrations were determined by a dot-blot assay with standard DIG-labeled RNA (100 ng/μl) (Roche). Excised gills were fixed with 4% paraformaldehyde overnight at 4°C and then washed several times with PBS. For the cryosections, fixed samples were immersed in PBS containing 30% sucrose overnight, and embedded in Optimal Cutting Temperature (OCT) compound embedding medium (Sakura, Tokyo, Japan) at -20°C, and 10-μm frozen cross-sections were cut with a CM 1900 rapid sectioning cryostat (Leica, Heidelberg, Germany) and attached to poly-L-lysine coated slides (Electron Microscopy Sciences, Ft. Washington, PA, USA). Prepared samples from either cryosections or methanol-dehydrated whole gills were washed several times with phosphate-buffered saline with 0.1% tween-20 (PBST). After a brief washing with PBST, samples were incubated with hybridization buffer (HyB) containing 50% formamide, 5× SSC, and 0.1% Tween-20 for 5 min at 65°C. Prehybridization was performed for 2 h at 65°C with HyB+, which is the hybridization buffer supplemented with 500 ng/mL yeast tRNA and 50 μg/mL heparin. For hybridization, samples were incubated in 100 ng of the RNA probe in 200 μL HyB+ at 65°C overnight. Then, slides were washed at 65°C for 10 min in 75% HyB and 25% 2× SSC, 10 min in 50% HyB and 50% 2× SSC, 10 min in 25% HyB and 75% 2× SSC, 10 min in 2× SSC, and 30 min for 2 times in 0.2× SSC at 70°C. Further washes were performed at room temperature for 5 min in 75% 0.2× SSC and 25% PBST, 5 min in 50% 0.2× SSC and 50% PBST, 5 min in 25% 0.2× SSC and 75% PBST, and 5 min in PBST. Fluorescence staining was conducted with a commercial kit, TSA Plus Fluorescence Systems (Perkin-Elmer). The hybridization signals detected by the DIG-labeled RNA probes were amplified through fluorescein-tyramide signal amplification (TSA), while cyanine 3-TSA was used for the DNP-labeled probes. For triple labeling, samples were first subjected to *in situ *hybridizations of the 2 transporter genes and then to Na^+^/K^+^-ATPase immunocytochemistry (see below).

### Immunohistochemistry

Zebrafish embryos and gills were fixed in 4% paraformaldehyde for 10 min at 4°C. After washing in PBS, fixed embryos were treated with 100% ethanol for 10 min at -20°C and subsequently subjected to blocking with 3% BSA at room temperature for 30 min. Embryos were then incubated with 1:200 PBS-diluted mouse anti-chicken Na^+^/K^+^-ATPase α subunit (cytosolic epitope for all isoforms) monoclonal immunoglobulin G (IgG) (Developmental Studies Hybridoma Bank, University of Iowa, Iowa City, IA, USA) at room temperature for 2 h. Samples were washed twice in PBS for 10 min each, and then incubated with 1:200 PBS-diluted goat anti-mouse IgG conjugated with Cy5 (Jackson Immunoresearch Laboratories, West Grove, PA, USA) for 2 h at room temperature. Images were acquired with a confocal laser scanning microscope (TCS-SP5, Leica Lasertechnik, Heidelberg, Germany).

### Quantitative real-time PCR

Quantitative real-time PCR (qPCR) was carried out using a SYBR green dye (Applied Biosystems)-based assay with an ABI Prism 7000 Sequence Detection System (Applied Biosystems) according to the manufacturer's instructions. Primers targeting Ca^2+ ^transporters (see additional file 1) and the endogenous control gene, beta-actin, were designed using the Primer Express 2.0 software (Applied Biosystems). In each assay, 25 ng cDNA was amplified in a 20-μL reaction containing 2x SYBR green master mix, 100 nM of forward and reverse primers, and nuclease-free water. Beta-actin, with a 151-bp amplicon (forward 5'-CACCTTCCAGCAGATGTGGA-3' and reverse 5'-AAAAGCCATGCCAATGTTGTC-3'), was used as an internal control to construct the standard curves.

### Statistical analysis

Values are presented as the mean ± SE and were compared by the 2-sample *t*-test.

## Abbreviations

BSA, bovine serum albumin; Dig, digoxigenin; DNP, dinitrophenol; ECaC, epithelium Ca^2+ ^channel; EST, expressed sequence tag; FW, freshwater; HCa, high Ca^2+^; HyB, hybridization buffer; LCa, low Ca^2+^; MR, mitochondrion-rich; NCX, Na^+^/Ca^2+ ^exchanger; OCT, Optimal Cutting Temperature; PBS, phosphate-buffered saline; PBST, phosphate-buffered saline with 0.1% tween-20; PMCA, plasma membrane Ca^2+^-ATPase; PNA, peanut lectin agglutinin; qPCR, Quantitative real-time PCR; RACE, rapid amplification of cDNA ends; RT-PCR, reverse-transcriptase polymerase chain reaction; SE, standard error; SERCA, sarco-endoplasmic reticulum calcium ATPase; SSC, SSC, saline-sodium citrate; TRP, transient receptor potential cation channel; TSA, fluorescein-tyramide signal amplification; UTR, untranslated regions; WGD, whole-genome duplication.

## Authors' contributions

PPH, the correspondence author, organized the whole project and manuscript. BKL designed and conducted the experiments and the data analysis; AND performed molecular cloning experiments; SCC, and MYC performed other biochemical experiments. All authors have read and approved the final manuscript.

## Supplementary Material

Additional file 1 Table S1: Primers for quantitative real-time PCR and the chromosome loci of zebrafish plasma membrane Ca^2+^-ATPase (PMCA) and Na^+^/Ca^2+ ^exchanger (NCX). The chromosome number and loci were obtained by the SSAHA program from the Ensembl database (zebrafish genome assemble version 6). Click here for file

## References

[B1] Hoenderop JG, Nilius B, Bindels RJ (2005). Calcium absorption across epithelia. Physioll Rev.

[B2] Flik G, Verbost PM, Wendelaar Bonga SE, Wood CM, Shuttleworth TJ (1995). Calcium transport process in fishes. Cellular and molecular approaches to fish ionic regulation.

[B3] Hwang PP, Tung YC, Chang MH (1996). Effect of environmental calcium levels on calcium uptake in tilapia larvae (*Oreochromis mossambicus*). Fish Physiol Biochem.

[B4] Pan TC, Liao BK, Huang CJ, Lin LY, Hwang PP (2005). Epithelial Ca^2+ ^channel expression and Ca(2+) uptake in developing zebrafish. Am J Physiol Regul Integr Comp Physiol.

[B5] Hwang PP, Tsai YN, Tung YC (1994). Calcium Balance in Embryos and Larvae of the Fresh-Water-Adapted Teleost, *Oreochromis-Mossambicus*. Fish Physio Biochem.

[B6] Flik G, Verbost PM, Wendelaar Bongar SE (1995). Calcium transport process in fishes Cellular and molecular approaches to fish ionic regulation.

[B7] Qiu A, Hogstrand C (2004). Functional characterisation and genomic analysis of an epithelial calcium channel (ECaC) from pufferfish, *Fugu rubripes*. Gene.

[B8] Shahsavarani A, McNeill B, Galvez F, Wood CM, Goss GG, Hwang PP, Perry SF (2006). Characterization of a branchial epithelial calcium channel (ECaC) in freshwater rainbow trout (*Oncorhynchus mykiss*). J Exp Biol.

[B9] Verbost PM, Schoenmakers TJ, Flik G, Wendelaar Bonga SE (1994). Kinetics of ATP- and Na^+^-gradient driven Ca^2+ ^transport in basolateral membranes from gills of freshwater- and seawater-adapted tilapia. J Exp Biol.

[B10] van der Heijden AJ, Verbost PM, Bijvelds MJ, Atsma W, Wendelaar Bonga SE, Flik G (1999). Effects of sea water and stanniectomy on branchial Ca^2+ ^handling and drinking rate in eel (*Anguilla anguilla *L.). J Exp Biol.

[B11] Shull GE (2000). Gene knockout studies of Ca^2+^-transporting ATPases. Eur J Biochem.

[B12] Strehler EE, Zacharias DA (2001). Role of alternative splicing in generating isoform diversity among plasma membrane calcium pumps. Physiol Rev.

[B13] Guerini D (1998). The significance of the isoforms of plasma membrane calcium ATPase. Cell Tissue Res.

[B14] Blaustein MP, Lederer WJ (1999). Sodium/calcium exchange: its physiological implications. Physiol Rev.

[B15] Wakimoto K, Kobayashi K, Kuro OM, Yao A, Iwamoto T, Yanaka N, Kita S, Nishida A, Azuma S, Toyoda Y (2000). Targeted disruption of Na^+^/Ca^2+ ^exchanger gene leads to cardiomyocyte apoptosis and defects in heartbeat. J Biol Chem.

[B16] Hoenderop JG, Dardenne O, Van Abel M, Van Der Kemp AW, Van Os CH, St-Arnaud R, Bindels RJ (2002). Modulation of renal Ca^2+ ^transport protein genes by dietary Ca^2+ ^and 1,25-dihydroxyvitamin D_3 _in 25-hydroxyvitamin D_3_-1alpha-hydroxylase knockout mice. Faseb J.

[B17] Marshall CR, Fox JA, Butland SL, Ouellette BF, Brinkman FS, Tibbits GF (2005). Phylogeny of Na^+^/Ca^2+ ^exchanger (NCX) genes from genomic data identifies new gene duplications and a new family member in fish species. Physiol Genomics.

[B18] Shu X, Huang J, Dong Y, Choi J, Langenbacher A, Chen JN (2007). Na,K-ATPase alpha2 and Ncx4a regulate zebrafish left-right patterning. Development.

[B19] Jaillon O, Aury JM, Brunet F, Petit JL, Stange-Thomann N, Mauceli E, Bouneau L, Fischer C, Ozouf-Costaz C, Bernot A (2004). Genome duplication in the teleost fish Tetraodon nigroviridis reveals the early vertebrate proto-karyotype. Nature.

[B20] Prince VE, Pickett FB (2002). Splitting pairs: the diverging fates of duplicated genes. Nat Rev.

[B21] Schulte PM (2004). Changes in gene expression as biochemical adaptations to environmental change: a tribute to Peter Hochachka. Comp Biochem Physiol B Biochem Mol Biol.

[B22] Hoenderop JG, van Leeuwen JP, van der Eerden BC, Kersten FF, van der Kemp AW, Merillat AM, Waarsing JH, Rossier BC, Vallon V, Hummler E (2003). Renal Ca^2+ ^wasting, hyperabsorption, and reduced bone thickness in mice lacking TRPV5. J Clin Invest.

[B23] Bijvelds M, Heijden A, Flik G, Verbost P, Kolar Z, Bonga S (1995). Calcium pump activities in the kidneys of *Oreochromis mossambicus*. J Exp Biol.

[B24] Hwang PP, Lee T (2007). New insights into fish ion regulation and mitochondria-rich cells. Comp Biochem Physiol A Mol Integr Physiol.

[B25] Langenbacher AD, Dong Y, Shu X, Choi J, Nicoll DA, Goldhaber JI, Philipson KD, Chen JN (2005). Mutation in sodium-calcium exchanger 1 (NCX1) causes cardiac fibrillation in zebrafish. P Natl Acad Sci USA.

[B26] Philipson KD, Nicoll DA (2000). Sodium-calcium exchange: a molecular perspective. Annu Rev Physiol.

[B27] Verma AK, Filoteo AG, Stanford DR, Wieben ED, Penniston JT, Strehler EE, Fischer R, Heim R, Vogel G, Mathews S (1988). Complete primary structure of a human plasma membrane Ca^2+ ^pump. J Biol Chem.

[B28] Iwamoto T, Nakamura TY, Pan Y, Uehara A, Imanaga I, Shigekawa M (1999). Unique topology of the internal repeats in the cardiac Na^+^/Ca^2+ ^exchanger. FEBS letters.

[B29] Nicoll DA, Ottolia M, Lu L, Lu Y, Philipson KD (1999). A new topological model of the cardiac sarcolemmal Na^+^-Ca^2+ ^exchanger. J Biol Chem.

[B30] Yamoah EN, Lumpkin EA, Dumont RA, Smith PJ, Hudspeth AJ, Gillespie PG (1998). Plasma membrane Ca^2+^-ATPase extrudes Ca^2+ ^from hair cell stereocilia. J Neurosci.

[B31] Kozel PJ, Friedman RA, Erway LC, Yamoah EN, Liu LH, Riddle T, Duffy JJ, Doetschman T, Miller ML, Cardell EL (1998). Balance and hearing deficits in mice with a null mutation in the gene encoding plasma membrane Ca^2+^-ATPase isoform 2. J Biol Chem.

[B32] Armbrecht HJ, Boltz MA, Kumar VB (1999). Intestinal plasma membrane calcium pump protein and its induction by 1,25-OH_2_D_3 _decrease with age. Am J Physiol.

[B33] Cai Q, Chandler JS, Wasserman RH, Kumar R, Penniston JT (1993). Vitamin D and adaptation to dietary calcium and phosphate deficiencies increase intestinal plasma membrane calcium pump gene expression. P Natl Acad Sci USA.

[B34] Zelinski JM, Sykes DE, Weiser MM (1991). The effect of vitamin D on rat intestinal plasma membrane Ca-pump mRNA. Biochem Bioph Res Co.

[B35] Shahsavarani A, Perry SF (2006). Hormonal and environmental regulation of epithelial calcium channel in gill of rainbow trout (*Oncorhynchus mykiss*). Am J Physiol Regul Integr Comp Physiol.

[B36] Evans DH, Piermarini PM, Choe KP (2005). The multifunctional fish gill: dominant site of gas exchange, osmoregulation, acid-base regulation, and excretion of nitrogenous waste. Physiol Rev.

[B37] Galvez F, Wong D, Wood CM (2006). Cadmium and calcium uptake in isolated mitochondria-rich cell populations from the gills of the freshwater rainbow trout. Am J Physiol Regul Integr Comp Physiol.

[B38] Hsiao CD, You MS, Guh YJ, Ma M, Jiang YJ, Hwang PP (2007). A Positive Regulatory Loop between foxi3a and foxi3b is Essential for Specification and Differentiation of Zebrafish Epidermal Ionocytes. PLoS ONE.

[B39] Prasad V, Okunade G, Liu L, Paul RJ, Shull GE (2007). Distinct phenotypes among plasma membrane Ca^2+^-ATPase knockout mice. Ann NY Acad Sci.

[B40] Reppel M, Sasse P, Malan D, Nguemo F, Reuter H, Bloch W, Hescheler J, Fleischmann BK (2007). Functional expression of the Na^+^/Ca^2+ ^exchanger in the embryonic mouse heart. J Mol Cell Cardiol.

[B41] Wasserman RH, Smith CA, Brindak ME, De Talamoni N, Fullmer CS, Penniston JT, Kumar R (1992). Vitamin D and mineral deficiencies increase the plasma membrane calcium pump of chicken intestine. Gastroenterology.

[B42] Pannabecker TL, Chandler JS, Wasserman RH (1995). Vitamin-D-dependent transcriptional regulation of the intestinal plasma membrane calcium pump. Biochem Bioph Res Co.

[B43] Marshall WS, Bryson SE (1998). Transport mechanisms of seawater teleost chloride cells: an inclusive model of a multifunctional cell. Comp Biochem Physiol A Mol Integr Physiol.

[B44] Srivastav AK, Flik G, Wendelaar Bonga SE (1998). Plasma calcium and stanniocalcin levels of male tilapia, *Oreochromis mossambicus*, fed calcium-deficient food and treated with 1,25 dihydroxyvitamin D_3_. Gen Compa Endocr.

[B45] Verbost PM, Flik G, Fenwick JC, Greco AM, Pang PKT, Bonga SEW (1993). Branchial Calcium-Uptake – Possible Mechanisms of Control by Stanniocalcin. Fish Physiol Biochem.

[B46] Verbost PM, Butkus A, Atsma W, Willems P, Flik G, Bonga SE (1993). Studies on stanniocalcin: characterization of bioactive and antigenic domains of the hormone. Mol Cell Endocrinol.

[B47] Vennekens R, Hoenderop JG, Prenen J, Stuiver M, Willems PH, Droogmans G, Nilius B, Bindels RJ (2000). Permeation and gating properties of the novel epithelial Ca^2+ ^channel. J Biol Chem.

[B48] Chang IC, Lee TH, Yang CH, Wei YY, Chou FI, Hwang PP (2001). Morphology and function of gill mitochondria-rich cells in fish acclimated to different environments. Physiol Biochem Zool.

[B49] Chen YY, Lu FI, Hwang PP (2003). Comparisons of calcium regulation in fish larvae. J Exp Zool.

[B50] Flik G, Kaneko T, Greco AM, Li J, Fenwick JC (1997). Sodium dependent ion transporters in trout gills. Fish Physiol Biochem.

[B51] Kumar S, Tamura K, Nei M (2004). MEGA3: Integrated software for Molecular Evolutionary Genetics Analysis and sequence alignment. Briefings in bioinformatics.

[B52] Rice P, Longden I, Bleasby A (2000). EMBOSS: the European Molecular Biology Open Software Suite. Trends Genet.

[B53] Persson B, Argos P (1994). Prediction of transmembrane segments in proteins utilising multiple sequence alignments. J Mol Biol.

[B54] Hsiao CD, Tsai WY, Horng LS, Tsai HJ (2003). Molecular structure and developmental expression of three muscle-type troponin T genes in zebrafish. Dev Dyn.

